# Modified dose difference method for comparing dose distributions

**DOI:** 10.1120/jacmp.v13i2.3616

**Published:** 2012-03-08

**Authors:** Jino Bak, Jin Hwa Choi, Jae‐Sung Kim, Suk Won Park

**Affiliations:** ^1^ Department of Radiation Oncology, College of Medicine Chung‐Ang University Seoul Korea; ^2^ Department of Radiation Oncology Seoul National University Bundang Hospital Seoul Korea

**Keywords:** modified dose difference (MDdiff) evaluation, gamma evaluation, dose distribution, IMRT, patient‐specific quality assurance (QA)

## Abstract

The quantitative comparison of two‐dimensional dose distributions (e.g., calculated versus measured) has become a key issue in intensity‐modulated radiotherapy (IMRT) QA. We proposed a new evaluation method referred to as modified dose difference (MDdiff) evaluation. Hereinafter, features and effectiveness of the MDdiff evaluation method will be described. In this work, the formalism of MDdiff was defined by introducing a dimensionless parameter $\beta_{\;DG}^{\left(r\right)}({\vec{r}}_{r})$. A new formalism is compared to a gamma method, and the MDdiff and the gamma method are respectively applied to patient‐specific IMRT QA. The calculation of the evaluation of dose distributions was performed using a C++ program. Evaluations were performed by counting the number of data points satisfying $\text{MDdiff}\geq (1/2)\delta D^{\left(0\right)},\gamma \geq 1$. The evaluation result of dose distributions using the MDdiff method had the same tendency as the evaluation result using the gamma evaluation method. The modified dose difference tool also provides a quantitative method for comparing two dose distributions like the gamma evaluation. Furthermore, many problems of gamma evaluation are resolved.

PACS numbers: 87.55.km, 87.55.Qr

## I. INTRODUCTION

Dose distribution comparison is performed routinely in radiotherapy (e.g., patient‐specific quality assurance (QA)) using 2D/3D dosimeters for intensity‐modulated radiation therapy (IMRT).^(^ ^1^ ^–^ ^3^ ^)^ The quantitative comparison of two‐dimensional dose distributions (e.g., calculated versus measured) has become a key issue in multidimensional dosimetry with the implementation of intensity‐modulated radiotherapy (IMRT).^(^ ^1^ ^,^ ^2^ ^,^ ^4^ ^–^ ^10^ ^)^

Simple evaluation by superimposing isodose distributions can only highlight or indicate areas of disagreement, but does not allow specifying the level of agreement/disagreement in a quantitative way. The dose difference test, wherein the difference between two dose distributions is calculated point by point in a dose domain, is the most straightforward and intuitive method. However, a large dose difference in a high‐dose gradient region is of less clinical significance, since a small alignment error can translate into a big dose error.^(^ ^1^ ^,^ ^11^ ^)^

Several comparison methods have been developed based on various combinations of doses and spatial acceptance tolerances, including the simple dose difference test, the distance‐to‐agreement (DTA) test, the composite analysis for both dose difference and DTA, and the gamma index method and its variations.^(^ ^1^ ^–^ ^4^ ^,^ ^11^ ^–^ ^13^ ^)^ Each comparison method has its own advantages and disadvantages.

The DTA measure does not work in low‐dose gradient regions and, therefore, is often used in conjunction with a dose difference measure in composite analysis.^(^ ^4^ ^,^ ^14^ ^,^ ^15^ ^)^ The composite analysis works in both high‐ and low‐dose gradient regions. The comparison fails only when both dose difference and DTA criteria fail and, therefore, the test is qualitative (pass/fail) rather than quantitative.

The gamma index calculation has recently become a popular dose comparison method due to its ability to produce a quantitative measure based on both dose and spatial criteria.^(^ ^1^ ^–^ ^3^ ^,^ ^12^ ^,^ ^13^ ^,^ ^16^ ^)^ The disadvantage of the gamma index is that its value, although quantitative, is less clinically intuitive than the dose difference. One can easily understand a dose difference of 3 cGy or 3%, but a gamma index of 2 is hard to understand. In addition, the gamma index is signless and, thus, one cannot tell which dose distribution has a higher value at the comparison point. Another disadvantage of this method is that it is sensitive to dose grid resolution.^(^ ^2^ ^,^ ^3^ ^,^ ^12^ ^)^

In this paper, we proposed a new method referred to as modified dose difference (MDdiff) evaluation. The MDdiff evaluation is relatively simple and intuitive. Many problems of a conventional dose difference test and/or gamma index method are resolved by the MDdiff evaluation. The MDdiff evaluation method will be compared to a gamma method, which is generally used in clinics, and advantages thereof will be described. Furthermore, the two methods will be used in patient‐specific quality assurance (QA), and the results will be presented.

## II. MATERIALS AND METHODS

### A.1 MDdiff evaluation methods

The method presented in this article uses a comparison between measured and calculated dose distributions. For generalization, we will use the terms “evaluated” and “reference” to replace calculated and measured, respectively.^(2)^

The most straightforward and intuitive method, the dose difference method, has a problem in a high‐dose gradient region. To resolve this problem, a particular factor related to the dose gradient shall be considered as the denominator of the dose difference. Furthermore, the factor shall not affect the dose difference in a low‐dose gradient region.

Thus, a dimensionless parameter $\beta_{\;DG}^{\left(r\right)}({\vec{r}}_{r})$ is defined as follows:

(1) β(r)(r→r)DG=|∇D(r)(r→r)|□(DTA(0)δD(0))

where $D^{\left(r\right)}({\overset{\to }{r}}_{r})$ is a reference dose distribution, $\delta D^{\left(0\right)}$ is the dose difference criterion, and ${\text{DTA}}^{\left(0\right)}$ is the distance‐to‐agreement (DTA) criterion. And an index *r* means position vector and gradient defined in the reference dose distribution. The criteria $\delta D^{\left(0\right)}$ and ${\text{DTA}}^{\left(0\right)}$ are exactly the same as in the gamma evaluation method.

The definition of a modified dose difference (MDdiff) distribution is:

(2) $$MDdiff({\vec{r}}_{r})=\delta^{\left(M\right)}D({\vec{r}}_{r})=\frac{\delta D({\vec{r}}_{r})}{1+\beta^{\left(r\right)}{\;}_{DG}({\vec{r}}_{r})}$$

where

(3) $${\delta D({\vec{r}}_{r})=D^{\left(e\right)}({\vec{r}}_{e})-D^{\left(r\right)}({\vec{r}}_{r})\|}_{where}\;\;\;\;{\;}_{{\vec{r}}_{e}={\vec{r}}_{r}}=Ddiff({\vec{r}}_{r})$$

is the dose difference at ${\overset{\to }{r}}_{r}$. Here, $D^{\left(e\right)}({\overset{\to }{r}}_{e})$ is an evaluated dose distribution.

In the case where $\nabla D^{\left(r\right)}({\overset{\to }{r}}_{r})\to 0,\text{MDdiff}({\overset{\to }{r}}_{r})\approx \text{Ddiff}({\overset{\to }{r}}_{r})$. Furthermore, in the case of $\nabla D^{\left(r\right)}({\overset{\to }{r}}_{r})\to \text{large}$, a large dose difference occurring in a high‐dose gradient region is eliminated from the value of $\text{MDdiff}({\overset{\to }{r}}_{r})$.

**Figure 1:**
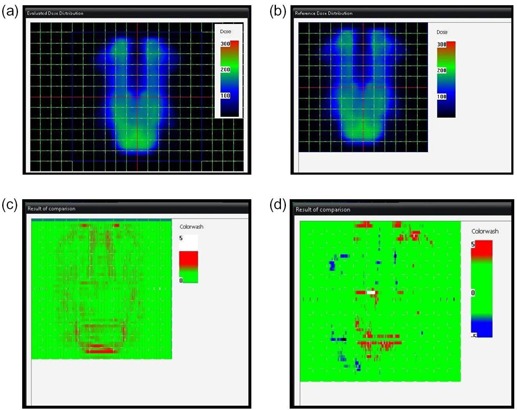
Pop‐up window of home‐made program for comparison of 2D dose distributions in IMRT QA: (a) reference dose distribution (cGy); (b) evaluated dose distribution (cGy); (c) gamma(unitless) distribution in gamma evaluation method; (d) MDdiff(cGy) distribution in MDdiff evaluation method. (The criteria are $\delta D^{\left(0\right)}=6\text{cGy}$ and ${\text{DTA}}^{\left(0\right)}=3\;\text{mm}$.)

While γ=1 is a critical value for pass/fail determination in gamma evaluation, $\text{MDdiff}=(1/2)\delta D^{\left(0\right)}$ is a corresponding critical value in MDdiff evaluation. $(1/2)\delta D^{\left(0\right)}$ is a value acquired from extensive experiments for patient‐specific IMRT quality assurance and fits with the result of gamma method. The known significant value of $\delta D^{\left(0\right)}$ is 3% of prescribed dose.^(^ ^1^ ^,^ ^4^ ^)^ In case of determining pass/fail by using $(1/2)\delta D^{\left(0\right)}$ as a critical value, similar results are acquired by using the gamma method and MDdiff method. Figure 1 shows an example of patient‐specific IMRT QA. The passing criteria are $\delta D^{\left(0\right)}=3\%$ of the prescribed dose and ${\text{DTA}}^{\left(0\right)}=3\;\text{mm}$ in calculations. This is the clinical standard for photon beams.^(^ ^1^ ^,^ ^4^ ^)^ The calculation of the evaluations was performed with an in‐house C++ program.

### B.1 Analysis of MDdiff evaluation and gamma evaluation

**Figure 2:**
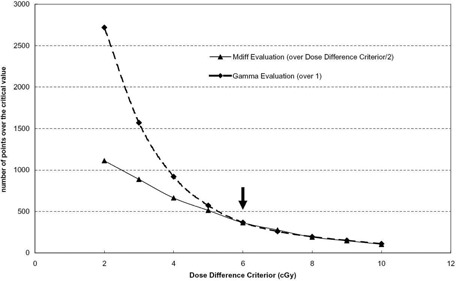
Results of the two evaluation methods (the number of points over the critical value vs. dose‐difference criterion, ${\text{DTA}}^{\left(0\right)}=3\;\text{mm})$.

**Figure 3:**
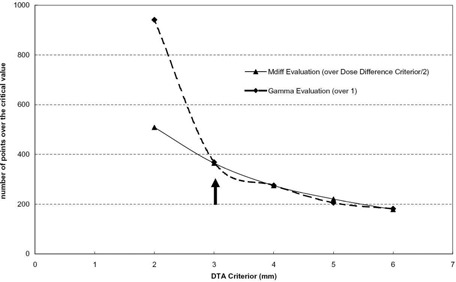
Results of the two evaluation methods (the number of points over the critical value vs. DTA criterion, $\delta D^{\left(0\right)}=6\;\text{cGy}=3\%$ of a prescribed dose).

Generally, the results of gamma evaluation and MDdiff evaluation depend on the two values — dose difference criterion $\left(\delta D^{\left(0\right)}\right)$ and DTA criterion $\left({\text{DTA}}^{\left(0\right)}\right)$. Figure 2 shows the results of each evaluation according to the variations of the dose difference criterion, and Fig. 3 shows results of each evaluation according to the variations of the DTA criterion. It is natural that the number of data points having values equal to or greater than a critical value decreases as the criteria increases.

Furthermore, a clinically meaningful point in Fig. 3 is the point corresponding to $\delta D^{\left(0\right)}=6\;\text{cGy}$ (3% of prescribed dose, thick arrow), whereas a clinically meaningful point in Fig. 3 is the point corresponding to ${\text{DTA}}^{\left(0\right)}=3\;\text{mm}$ (thick arrow). In QA plans, the prescribed dose is 200 cGy. At these points, the results of gamma evaluation and MDdiff evaluation are almost identical to each other.

**Figure 4:**
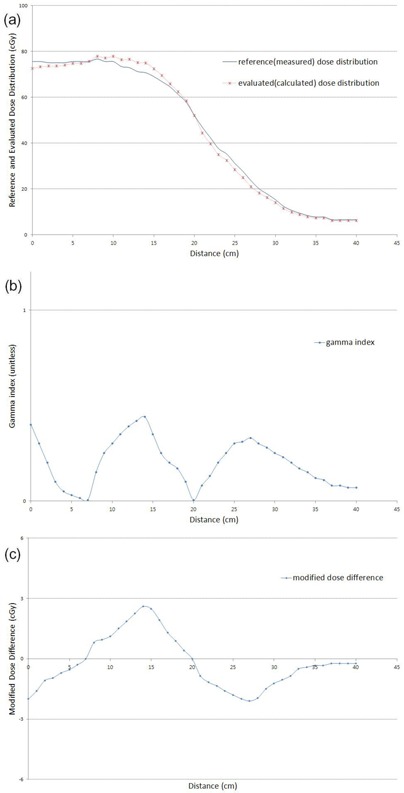
Measured (a) and calculated (b) dose distributions $\gamma ({\overset{\to }{r}}_{r})$, and $\text{MDdiff}({\overset{\to }{r}}_{r})$ (c) for the condition $\delta D^{\left(0\right)}=6\;\text{cGy}$ and ${\text{DTA}}^{\left(0\right)}=3\;\text{mm}$.

Figures 4(a) and (b) show the measured and calculated dose distributions, and the $\gamma ({\overset{\to }{r}}_{r})$, for the condition $\delta D^{\left(0\right)}=6\;\text{cGy}$ and ${\text{DTA}}^{\left(0\right)}=3\;\text{mm}$. Figure 4(c) shows the $\text{MDdiff}({\overset{\to }{r}}_{r})$ for the same measured and calculated dose distributions.

## III. RESULTS

Patient‐specific IMRT QA using a two‐dimensional dose distribution was performed. The planned dose distribution and the measured dose distribution were obtained, and were evaluated by using two methods (MDdiff, gamma). Evaluations were performed by counting the number of data points satisfying $\text{MDdiff}\geq (1/2)\delta D^{\left(0\right)},\;\gamma \geq 1$.

Actually, in our institution, patient‐specific IMRT QA was routinely performed. Patient‐specific IMRT QA was already performed 100 times or more. About 87% of all cases using the two methods had the same evaluation result. The evaluation results of dose distributions using the MDdiff method had the same tendency as the evaluation result using the gamma evaluation method. In other words, IMRT QA of patients with high coincidence indexes in the evaluation using the MDdiff method also had high coincidence indexes in the evaluation using the gamma evaluation method. The same tendency appeared with respect to patient‐specific IMRT QA with low coincidence indexes.

**Figure 5:**
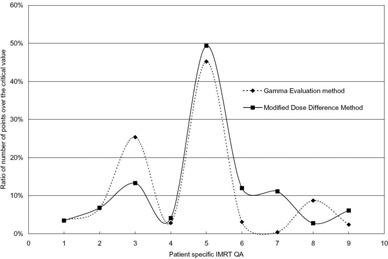
Tendency of two evaluation methods (ratio number of points over the critical value in nine IMRT QA).

Table 1 shows examples of data regarding nine patients. Figure 5 shows ratios of number of points over the critical value, for the two methods. In some cases (e.g., QA3), it appeared that there was a large difference between the results of the two methods. However, when many data points exist around a critical value $\left(\gamma =1,\;\text{MDdiff}=(1/2)\delta D^{\left(0\right)}\right)$, if pass/fail determination was performed based on the critical value, the results could be very sensitive.

**Table 1:** Results of patient‐specific IMRT QA. $({\text{DTA}}^{\left(0\right)}=3\%$ of prescribed dose, ${\text{DTA}}^{\left(0\right)}=3\;\text{mm})$.

*Patient‐specific IMRT QA*		*QA 1*	*QA 2*	*QA 3*	*QA 4*	*QA 5*	*QA 6*	*QA 7*	*QA 8*	*QA 9*
Prescribed Dose (cGy)		180	180	200	180	200	100	120	100	140
Total # of Data Points		10,560	5,760	10,120	7,920	7,744	4,672	6,664	2,880	8,000
	γ≥1	369	388	2569	226	3502	145	28	252	193
Gamma Evaluation	(ratio)	3.49%	6.74%	25.39%	2.85%	45.22%	3.10%	0.42%	8.75%	2.41%
	$\gamma_{\text{avg}}$	0.59	0.60	0.85	0.6	1.33	0.60	0.55	0.65	0.62
	$\gamma_{\max}$	10.71	2.25	37.28	1.5	6.64	1.79	1.45	2.93	2.35
	$\text{MDdiff}\geq (1/2)\delta D^{\left(0\right)}$	365	393	1,345	325	3,822	560	741	80	489
	(ratio)	3.46%	6.82%	13.29%	4.10%	49.35%	11.99%	11.12%	2.78%	6.11%
MDdiff Evaluation	${\text{MDdiff}}_{\text{avg}}(\text{cGy})$	0.10	0.60	−1.20	−0.25	5.86	0.32	0.97	−0.07	−0.21
	${\text{MDdiff}}_{\left|\text{avg}\right|}(\text{cGy})$	1.00	1.39	3.00	1.30	6.21	1.62	1.18	0.60	1.14
${\text{MDdiff}}_{\max}(\text{cGy})$	10.92	6.85	15.41	8.68	36.17	10.54	5.26	5.10	9.78

Additionally, the average values and maximum values of $\text{MDdiff}({\overset{\to }{r}}_{r})$ and $\gamma ({\overset{\to }{r}}_{r})$ in Table 1, which may be used as supplementary evidence, also had the same tendency in the two evaluation methods.

In actual clinical tests, if the ratio of $\text{MDdiff}\geq (1/2)\delta D^{\left(0\right)}$ is relatively large (e.g., QA5 in Table 1), the average value and maximum value of $\text{MDdiff}({\overset{\to }{r}}_{r})$ and the locations of points where $\text{MDdiff}({\overset{\to }{r}}_{r})$ is relatively large are considered for the final determination of a comparison result.

## IV. DISCUSSION & CONCLUSION

In this paper, we set forth a methodology and describe a tool that can be used in a simple and effective way to evaluate 2D dose distributions. An important benefit of this technique is simple formalism and intuitive result of evaluation.

In the gamma index method, the process of acquiring a gamma index distribution is highly complicated. If a number of data points of the dose distribution increases, the amount of required calculations massively increases and, thus, the calculations depend on the computer's parameters. For example, the number of calculations required is *mn* in the case of employing the gamma method for comparing a reference distribution with *m* data points and an evaluated distribution with *n* data points, whereas the number of calculations required for such comparison is *m* in the case of employing the MDdiff method. This has the potential to provide a powerful analysis tool in quantitative evaluation of three‐dimensional dose distributions. Considering that patient‐specific IMRT QA is developed from comparisons of 2D dose distributions to comparisons of 3D dose distributions, the MDdiff method may become more useful.

Furthermore, the MDdiff evaluation method suggested in the present research features intuitive and simple formalisms. Furthermore, a modified dose difference distribution, $\text{MDdiff}({\overset{\to }{r}}_{r})$, can be generated and displayed, providing a quantitative assessment of the quality of the calculation similarly to the gamma method. We believe that the modified dose difference concept set forth in this paper provides a valuable method for quantitative comparison of dose distributions.

Furthermore, although the gamma index is dimensionless, $\text{MDdiff}({\overset{\to }{r}}_{r})$ is a physical quantity of which unit is cGy. Therefore, one can easily understand these quantities. Additionally, $\text{MDdiff}({\overset{\to }{r}}_{r})$ in Fig. 1 has a sign and, thus, we can tell which dose distribution has a higher value at the comparison point.

## ACKNOWLEDGMENTS

This study was supported by the Chung‐Ang University Research Grants in 2009.
